# Hybrid Control of the DC Microgrid Using Deep Neural Networks and Global Terminal Sliding Mode Control with the Exponential Reaching Law

**DOI:** 10.3390/s23239342

**Published:** 2023-11-22

**Authors:** Mohamed A. Sharaf, Hammad Armghan, Naghmash Ali, Amr Yousef, Yasser S. Abdalla, Anis R. Boudabbous, Hafiz Mehdi, Ammar Armghan

**Affiliations:** 1 Department of Computer Engineering and Networks, College of Computer and Information Sciences, Jouf University, Sakaka 72388, Saudi Arabia; masharaf@ju.edu.sa (M.A.S.); ysabdalla@ju.edu.sa (Y.S.A.); aboudabbous@ju.edu.sa (A.R.B.); 2 Tsinghua Shenzhen International Graduate School, Tsinghua University, Shenzhen 518055, China; 14mseeharmghan@seecs.edu.pk (H.A.); 14mseenmr@seecs.edu.pk (N.A.); 3 Electrical Engineering Department, University of Business and Technology, Ar Rawdah, Jeddah 23435, Saudi Arabia; a.yousef@ubt.edu.sa; 4 Engineering Mathematics Department, Alexandria University, Lotfy El-Sied st. off Gamal Abd El-Naser, Alexandria 11432, Egypt; 5 School of Electrical Engineering and Computer Science (SEECS), National University of Science and Technology (NUST), Islamabad 44000, Pakistan; hmehdi.msee19seecs@seecs.edu.pk; 6 Department of Electrical Engineering, College of Engineering, Jouf University, Sakaka 72388, Saudi Arabia

**Keywords:** deep neural networks, maximum power point tracking, nonlinear controller, power converters, photovoltaic, wind turbine

## Abstract

The direct current (DC) microgrid is one of the key research areas for our advancement toward carbon-free energy production. In this paper, a two-step controller is designed for the DC microgrid using a combination of the deep neural network (DNN) and exponential reaching law-based global terminal sliding mode control (ERL-GTSMC). The DC microgrid under consideration involves multiple renewable sources (wind, PV) and an energy storage unit (ESU) connected to a 700 V DC bus and a 4–12 kW residential load. The proposed control method eliminates the chattering phenomenon and offers quick reaching time by utilizing the exponential reaching law (ERL). In the two-step control configuration, first, DNNs are used to find maximum power point tracking (MPPT) reference values, and then ERL-based GTSMC is utilized to track the reference values. The real dynamics of energy sources and the DC bus are mathematically modeled, which increases the system’s complexity. Through the use of Lyapunov stability criteria, the stability of the control system is examined. The effectiveness of the suggested hybrid control algorithm has been examined using MATLAB simulations. The proposed framework has been compared to traditional sliding mode control and terminal sliding mode control to showcase its superiority and robustness. Experimental tests based on the hardware-in-the-loop (HIL) setup are then conducted using 32-bit TMS320F28379D microcontrollers. Both MATLAB and HIL results show strong performance under a range of environmental circumstances and system uncertainties.

## 1. Introduction

Carbon emissions reached their highest level in 2021 after a 6% increase. This is mainly due to the excessive use of fossil fuels to meet our energy demands [1]. The utilization of renewable energy resources (RERs) is a promising way to generate clean energy and reduce carbon emissions [2,3]. RERs are also becoming a convenient way to provide electricity to rural areas [4]. We have seen significant improvements in the technologies related to renewable energy systems (RESs) during the last decade. Solar, hydro, wind, biofuel, and ocean energy are a few of the RERs that are available in abundance [5].

Solar and wind energy systems are the most utilized energy technologies [6,7]. However, climatic factors such as solar irradiance, temperature, air density, and wind speed have an impact on the generated power [8]. Hence, due to their intermittent nature, a combination of solar and wind to form a hybrid renewable energy system seems to be an effective way to provide reliable power [6,9]. Typically, an energy storage unit (ESU), such as a battery, ultracapacitor, or fuel cell, is attached to hybrid systems to store excess power and deliver power when RESs generated power is insufficient [10,11,12]. Since most of the RESs and ESU are direct current (DC) devices, the DC microgrid is emerging as one of the key research areas [13,14,15]. DC microgrids are efficient and also have the advantage of simple operation and robust control because of the use of semiconductor devices [11]. Moreover, RESs and ESU can be connected to the DC system without having to worry about synchronization, DC/AC conversions, or power quality concerns [15,16].

One of the major challenges in the study of DC microgrids is the stability of the DC bus voltage and the transfer of reliable power under varying weather conditions and system dynamics [17,18]. Different linear control methods for renewable-based DC microgrids are presented in the literature to stabilize the DC bus voltage. However, linear controllers have their limitations and cannot deal with the uncertainties present in the system [19,20,21,22]. A two-step energy management system and proportional-integral-derivative (PID) control for DC microgrid are proposed in [20]. The controller is simple and easy to implement but has some limitations such as slow reaching time, a small operating region, and an inability to handle nonlinearities that exist in the framework. A fuzzy-PID controller for wind and PV-based DC microgrid is discussed in [22]. Fuzzy logic is a model-free control technique and simple to implement, but since it depends mostly on human insight and expertise, it cannot recognize machine learning and neural networks. In one paper [21], a mathematical model of DC microgrid components and an energy storage system is developed, and model predictive control (MPC) is designed. Although the proposed controller is effective, MPC has a number of drawbacks, including the need for additional sensors and the excessive use of computational power. To overcome these drawbacks, based on higher order sliding mode control theory, several controllers [23,24] have been devised for DC microgrid but these exhibit the chattering phenomenon leading to lower accuracy.

The controllers described so far are based on linear control theory and can only operate in a small region. However, DC microgrids have nonlinear elements and uncertainties. Hence, the next step is to investigate the design of nonlinear controllers for DC microgrids. Nonlinear controllers are robust and have the ability to provide asymptotic stability, fast convergence, and a large region of operation [25]. Different nonlinear controllers, such as Lyapunov redesign, sliding mode control, and backstepping control, have been proposed for DC microgrids in the literature [18,26,27,28,29]. A nonlinear controller is designed to integrate PV panels in a DC microgrid setup, and its performance is compared with PI and vectorial control [18]. A backstepping-based decentralized nonlinear controller is formulated in [28], and the stability of the DC bus is tested. The controller gives satisfactory performance but suffers from calculation explosion. Furthermore, this reference generation in this control methodology utilizes linear regression which has lower accuracy. In [29], an energy management strategy and integral sliding mode control are proposed for a DC microgrid. The proposed control shows a chattering phenomenon and exhibits steady-state error.

Keeping in mind the preceding arguments, this work studies a DC microgrid comprised of a wind turbine, PV, and battery. A two-step low-level controller is designed with a DNN-based MPPT reference generation for renewable sources and exponential reaching law-based global terminal sliding mode control (ERL-GTSMC) for reference tracking. For reference generation, a DNN model is configured using the TensorFlow open-source Python package, and the data are exported to MATLAB/Simulink 2023a. DNN is utilized because of its sensorless operation, high accuracy, and ease of implementation [30]. For the tracking controller, ERL-GTSMC is proposed since it has a fast reaching time and forces the system to the equilibrium point in a finite amount of time, even when the system is not close to a sliding surface [17]. Furthermore, the chattering phenomenon found in sliding mode control is significantly reduced by ERL-GTSMC [31]. At the high-level control, the energy management algorithm is developed using the battery SOC. No other studies in the literature that the authors are aware of have addressed the ERL-based GTSMC for DC microgrid. The following are the primary contributions of the proposed study:

1. A two-step low-level controller based on DNN and ERL-GTSMC is developed for the wind/PV/battery DC microgrid.
2. A TensorFlow open-source Python library is utilized for DNN testing and training. A Pymgrid Python library is used to retrieve PV and load profiles.
3. Real-world weather profiles and residential building loads are used to test the performance of the controllers.
4. Using TMS320F2837D microcontrollers (Texas Instruments, Dallas, TX, USA) hardware-in-the-loop experiments are conducted to analyze the behavior of a controller in industrial scenarios.

The paper has been structured as follows: In Section 2, the mathematical description of all components of the DC microgrid is presented, and DNN models are discussed. Section 3 illustrates the design technique for the ERL-GTSMC-based tracking controllers. Section 4 discusses the energy management system of the proposed framework. The simulation and HIL results are discussed in Section 5. The article concludes with Section 6.

## 2. System Modeling and DNN Design

**Figure 1:**
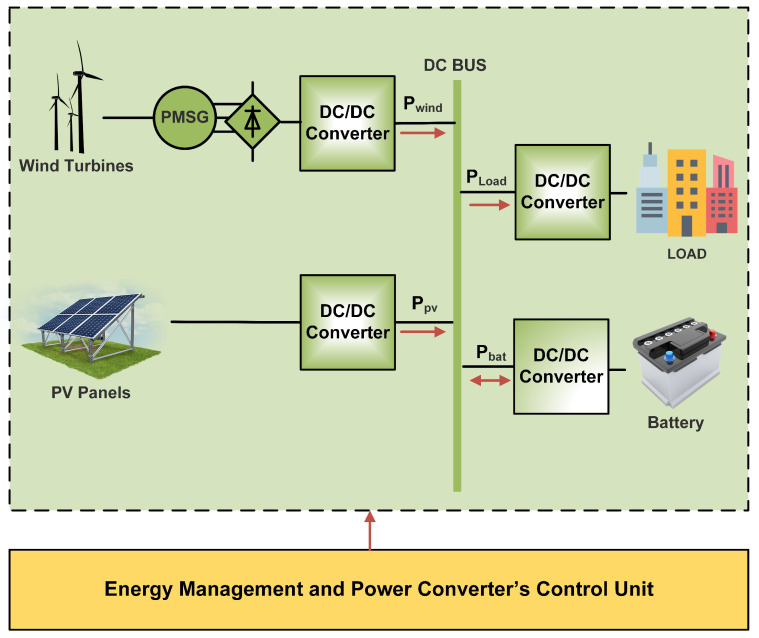
DC microgrid under study.

The overall block diagram of the proposed Wind/PV/Battery DC microgrid is depicted in Figure 1, where PV, wind energy systems, and ESU are linked with the microgrid DC bus via DC-DC power converters. A boost converter is connected to the wind energy system (WES) to control its power and provide MPPT. In addition, the buck-boost bidirectional converter has been utilized to control power flow to the ESU. The PV energy system (PVS) is energized through a two-switch non-inverted buck-boost converter. A varying local DC load is also connected to the microgrid.

### 2.1. Modeling of WES and DNN Design

**Figure 2:**
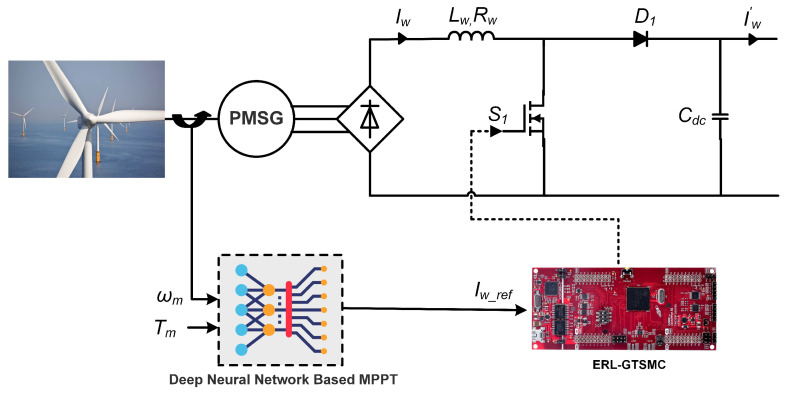
WES utilizing DNN and ERL-GTSMC controllers.

This research examines WES based on permanent magnet synchronous generators (PMSG) and wind turbines. As seen in Figure 2, a DC-DC boost converter, along with an unregulated rectifier, has been used to link the PMSG with the DC bus. Due to its simplicity, low cost, and ease of management, this study favors PMSG-based wind turbines. By utilizing generators and turbines, WES converts wind energy into electrical energy. The following mathematical equation can be used to express the mechanical power produced by the WES [32]:

(1) $$P_{mech}=\frac{1}{2}\rho \pi r_{w}^{2}v_{w}^{3}C_{m}\left(\lambda ,\beta \right),$$

where $v_{w}$, ρ, λ, β, and $r_{w}$ represent wind speed, air density, tip speed ratio, blade pitch angle, and turbine radius, respectively. Furthermore, $C_{p}$ represents the power coefficient derived in the form of (λ) and (β). In addition, λ and shaft speed $\omega_{r}$ have been related by the expression [33]:

(2) $$\lambda =\frac{\omega_{r}r_{w}}{v_{w}},$$

where $\omega_{r}$ represents the rotor angular velocity. By utilizing Equations (1) and (2), $P_{mech}$ can be derived as follows,

(3) $$P_{mech}=\frac{1}{2}\rho \pi r_{w}^{5}C_{m}\frac{\omega_{r}^{3}}{\lambda^{3}}.$$

**Figure 3:**
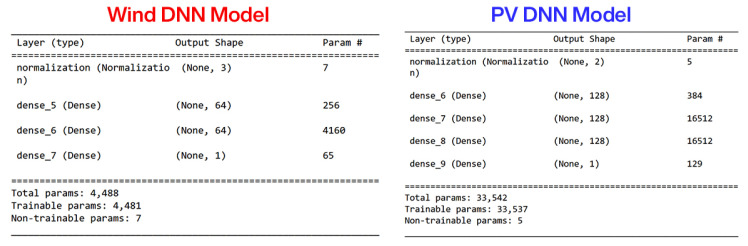
Configuration of DNN for WES and PVS.

For MPPT of the WES, DNN is used for the reference generation. DNN is employed because of its increased precision and sensorless operation. The DNN consists of one input layer with two neurons, 2 hidden layers with 64 neurons each, and one output layer with one neuron. In this DNN, 54,003 data points for training and testing are utilized. The details of the DNN are given in Figure 3. Data for offline training to learn the characteristics of the wind turbine as a function of inductor current $\left(I_{w}\right)$, shaft speed, and mechanical torque are obtained using a mathematical equation-based OTC. For a specific wind speed, the OTC method operates by regulating PMSG torque to the wind turbine’s maximum reference torque $T_{mech}^{*}$. Under the assumption that the rotor is operating at an ideal tip-speed ratio $\lambda_{m}$ and maximum power coefficient $C_{p-m}$, (3) can be modified as,

(4) $$P_{mech-m}=\frac{1}{2}\rho \pi {r_{w}}^{5}\omega_{r}^{3}\frac{C_{p-m}}{\lambda_{m}^{3}}=K_{p-m}\omega_{r}^{3}.$$

Since $P_{mech-m}=\omega_{m}T_{mech}^{*}$, we can rewrite (4) as:

(5) $$T_{mech}^{*}=\frac{1}{2}\rho \pi r_{w}^{5}\frac{C_{p-m}}{\lambda_{m}^{3}}\omega_{r}^{2}=K_{p-m}\omega_{r}^{2}.$$

Calculating $T_{mech}^{*}$ from (5), the reference current $I_{wref}$ can be derived as [33]:

(6) $$I_{wref}=\frac{T_{mech}^{*}\times \omega_{r}}{V_{wind}},$$

where the WES power converter’s input voltages ($V_{wind}$) serve as an apparent load to regulate PMSG rotating speed. The converter is made up of an IGBT switch $S_{1}$, an output filter capacitor $C_{dc}$, an input inductor $L_{w}$, and a diode $D_{1}$. Based on literature presented in [17], the mathematical model of WES can be derived using state–space as follows:

(7) $$\left\{\begin{array}{c} \left[\begin{array}{c} \frac{di_{w}}{dt}\\ \frac{dv_{dc}}{dt} \end{array}\right]=\left[\begin{array}{cc} \frac{R_{w}}{L_{w}} & \frac{-(1-\mu_{1})}{L_{w}}\\ \frac{\left(1-\mu_{1}\right)}{C_{dc}} & -\frac{I_{w}^{{}^{\prime }}}{v_{dc}C_{dc}} \end{array}\right]\cdot \left[\begin{array}{c} i_{w}\\ v_{dc} \end{array}\right]+\left[\begin{array}{c} \frac{1}{L_{w}}\\ 0 \end{array}\right]V_{w},\\ y=\left[\begin{array}{cc} 0 & 1 \end{array}\right]\cdot \left[\begin{array}{c} i_{w}\\ v_{dc} \end{array}\right], \end{array}\right.$$

where $i_{w}$, $\mu_{1}$ and $v_{dc}$ are the wind current, switching control, and output voltage, respectively.

### 2.2. Modeling of PVS and DNN Design

**Figure 4:**
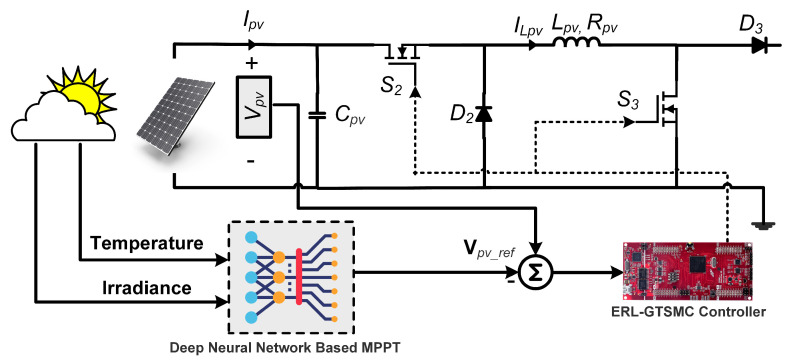
PV energy system with DNN and ERL-GTSMC controller.

The PVS presented in Figure 4 is comprised of PV modules interfaced via a DC-DC non-inverting buck-boost converter. The MPPT operation is carried out by the DC-DC converter, which tracks the DNN-generated MPP voltage reference. The DNN architecture involves an input layer with two neurons, three hidden layers with 128 neurons each, and an output layer with one neuron. 630,083 data points are used for the DNN training and testing. Additionally, the offline training data of the MPP reference voltage as a function of temperature and irradiance is obtained using the following equation [34]:

(8) $$V_{pv_{ref}}=322-\left(1.34\times Temperature\right)-\left(0.00964\times Irradiance\right).$$

The PVS converter is composed of two insulated gate bipolar transistor (IGBT) switches $\left(S_{2},S_{3}\right)$, an inductor $L_{pv}$, a PV input capacitor $\left(C_{pv}\right)$, two diodes $\left(D_{2},D_{3}\right)$, and a PV output capacitor $C_{dc}$. The proposed model also considers the converter’s continuous conduction operation. The DC-DC converter has two operating modes. In mode 1, the PV system is disconnected from the load side, $D_{3}$ is reverse biased, and the IGBT switches are ON. In mode 2, both IGBT switches are OFF, and a forward-biased diode will connect the load side of the inductor ($L_{pv}$) to the circuit.

The governing differential equations of PVS can be expressed as follows:

(9) $$\left\{\begin{array}{c} \left[\begin{array}{c} \frac{dV_{pv}}{dt}\\ \frac{di_{Lpv}}{dt}\\ \frac{dv_{dc}}{dt} \end{array}\right]=\left[\begin{array}{ccc} \frac{1}{C_{pv}} & \frac{\left(-\mu_{2}\right)}{C_{pv}} & 0\\ 0 & 0 & \left(\frac{\mu_{2}}{L_{pv}}-\frac{1}{L_{pv}}\right)\\ 0 & \left(\frac{1}{C_{dc}}-\frac{\mu_{2}}{C_{dc}}\right) & \frac{{I_{pv}}^{{}^{\prime }}}{v_{dc}C_{dc}} \end{array}\right]\cdot \left[\begin{array}{c} I_{pv}\\ i_{Lpv}\\ v_{dc} \end{array}\right]+\left[\begin{array}{c} 0\\ \frac{\left(\mu_{2}\right)}{L_{pv}}\\ 0 \end{array}\right]V_{pv},\\ y=\left[\begin{array}{ccc} 0 & 0 & 1 \end{array}\right]\cdot \left[\begin{array}{c} I_{pv}\\ i_{Lpv}\\ v_{dc} \end{array}\right], \end{array}\right.$$

where $V_{pv}$, $\mu_{2},$ and $i_{Lpv},$ are the input capacitor voltages, control signal, and inductor current of the PV system, respectively.

### 2.3. Modeling of Battery

**Figure 5:**
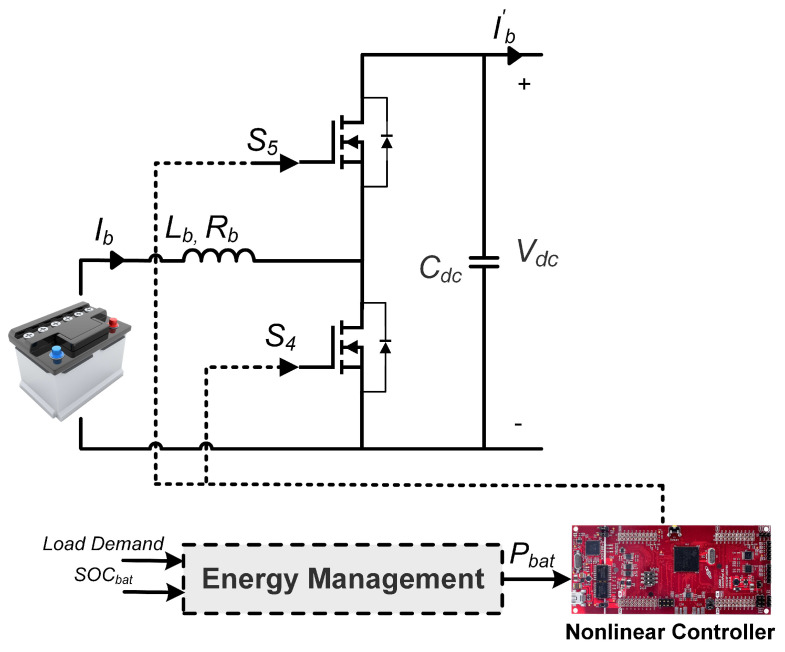
Battery with ERL-GTSMC controller.

Standard lead-acid batteries are taken into consideration in this study due to their accessibility and affordability. As shown in Figure 5, a two-switch DC-DC buck-boost converter connects the battery to the DC bus and allows bi-directional current flow. The battery’s job is to regulate the DC bus voltage at a specific reference while the power to the load and generation from WES and PVS are changing. The State of Charge (SOC) of the battery can be determined as follows [35]:

(10) $$SOC=100\left(1+\frac{\int I_{bat}dt}{Q}\right)$$

A crucial factor in the Energy Management System (EMS) is the battery’s State of Charge (SOC), which measures how much battery power is remaining compared to the maximum amount of power it can store. The EMS needs to receive the battery’s SOC to determine whether to charge or discharge the battery based on the amount of load demand. A battery’s nominal capacity (*Q*) and charging current ($I_{bat}$) influence the number of amp-hours it can store over a specific time interval (*t*). The following SOC limitations are necessary for a battery to function safely and have a long lifespan:

(11) $$SOC_{\min}\leq SOC\leq SOC_{\max},$$

where $SOC_{\min}$ and $SOC_{\max}$ represent the lowest and highest permissible states for the safe operation of the battery. Furthermore, the state-space model of the connected converter can be described as follows:

(12) $$\left\{\begin{array}{c} \left[\begin{array}{c} \frac{di_{b}}{dt}\\ \frac{dv_{dc}}{dt} \end{array}\right]=\left[\begin{array}{cc} \frac{R_{b}}{L_{b}} & \frac{-\mu_{34}}{L_{fc}}\\ \frac{\mu_{34}}{C_{dc}} & -\frac{I_{b}^{{}^{\prime }}}{v_{dc}C_{dc}} \end{array}\right]\cdot \left[\begin{array}{c} i_{b}\\ v_{dc} \end{array}\right]+\left[\begin{array}{c} \frac{1}{L_{b}}\\ 0 \end{array}\right]V_{b},\\ y=\left[\begin{array}{cc} 0 & 1 \end{array}\right]\cdot \left[\begin{array}{c} i_{b}\\ v_{dc} \end{array}\right], \end{array}\right.$$

where $I_{b}$, $V_{b}$ are the battery current and voltage. Furthermore, $\mu_{34}$ represents the switching control that can be defined as follows:

(13) $$\begin{array}{cc} \; & \mu_{34}=\left(1-j\right)d_{4}+j\left(1-d_{3}\right)\\ \; & j=\left\{\begin{array}{cc} 1 & I_{b}^{*}>0\;(\mathrm{Discharging}\;\mathrm{Mode})\\ 0 & I_{b}^{*}<0\;(\mathrm{Charging}\;\mathrm{Mode}) \end{array}\right. \end{array}$$

### 2.4. Microgrid Global Modeling

By merging the mathematical models presented in Equations (7), (9) and (12), the proposed DC microgrid global mathematical model based on differential equations can be stated as follows:

(14) $$\frac{dx_{1}}{dt}=\frac{V_{w}}{L_{w}}-\frac{R_{w}x_{1}}{L_{w}}-\frac{x_{5}\left(1-\mu_{1}\right)}{L_{w}},$$

(15) $$\frac{dx_{2}}{dt}=\frac{I_{pv}}{C_{dc}}-\frac{\mu_{2}x_{3}}{C_{dc}},$$

(16) $$\frac{dx_{3}}{dt}=\frac{x_{2}\mu_{2}}{L_{pv}}-\frac{x_{5}\left(1-\mu_{2}\right)}{L_{pv}},$$

(17) $$\frac{dx_{4}}{dt}=\frac{V_{b}}{L_{b}}-\frac{R_{b}x_{4}}{L_{b}}-\frac{x_{5}\mu_{34}}{L_{b}},$$

(18) $$\begin{array}{c} \begin{array}{c} \frac{dx_{5}}{dt}=\left(1-\mu_{1}\right)\frac{x_{1}}{C_{dc}}+\left(1-\mu_{2}\right)\frac{x_{3}}{C_{dc}}+\mu_{34}\frac{x_{4}}{C_{dc}}-\frac{I_{o}}{C_{dc}}, \end{array} \end{array}$$

where $x_{1},x_{2},x_{3}$, $x_{4}$ and $x_{5}$ represent average values of $i_{w}$, $V_{pv}$, $i_{Lpv}$, $i_{b}$ and $v_{dc}$, respectively. Moreover, $I_{o}$ represents the load current.

## 3. Design of ERL-GTSMC (Low-Level)

**Figure 6:**
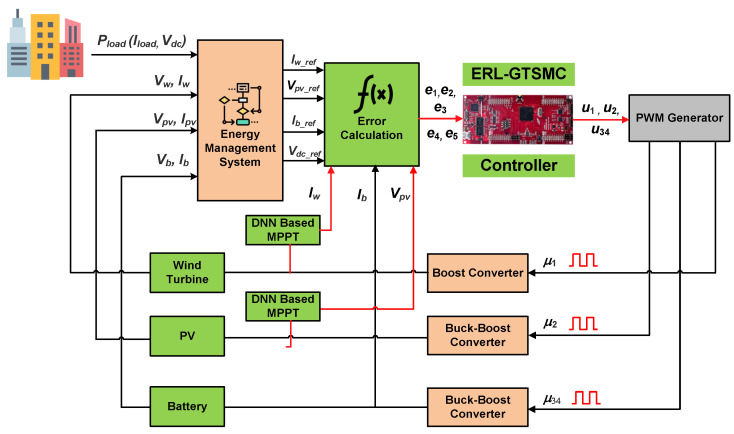
Overview of the ERL-GTSMC based low-level controllers.

Based on ERL-GTSMC multiple controllers have been designed for PV/wind/battery DC microgrid. The centralized control framework for the microgrid has been shown in Figure 6. The core objectives for the switching control laws are:

- MPPT for WES and PVS by tracking current and voltage references provided by DNN
- Tracking of Battery power to satisfy load demand
- Tracking of DC bus voltage under deviating load demand and weather conditions

For the ERL-GTSMC controllers design, the error signals have been defined:

(19) $$e=\left[\begin{array}{c} e_{1}\\ e_{2}\\ e_{3}\\ e_{4} \end{array}\right]=\left[\begin{array}{c} x_{1}-{I_{w}}^{*}\\ x_{2}-{V_{pv}}^{*}\\ x_{4}-{I_{b}}^{*}\\ x_{5}-{V_{dc}}^{*} \end{array}\right],$$

where ${I_{w}}^{*},{V_{pv}}^{*},{I_{b}}^{*}$, and ${V_{dc}}^{*}$ represent the reference values generated by the EMS, devised in Section 4. Based on the GTSMC theory, the sliding surface $S_{1}$ for the minimization of $e_{1}$ to zero has been derived as:

(20) $$S_{1}=e_{1}+\eta_{1}\int_{0}^{t}e_{1}\cdot dt+\lambda_{1}{\left(\int_{0}^{t}e_{1}\cdot dt\right)}^{\theta_{1}},$$

where $\eta_{1}>0$, $\lambda_{1}>0$, $0<\theta_{1}<2$, and $\theta_{1}=\frac{p_{1}}{q_{1}}$, and both $p_{1}$ and $q_{1}$ are positive odd numbers. By tuning these control parameters, not only can chattering be reduced but fast tracking can also be achieved. Utilizing (20) and (14), the dynamics of the sliding surface (${\dot{S}}_{1}$) can be derived by taking the time derivative:

(21) $$\dot{S_{1}}=\frac{V_{w}}{L_{w}}-\frac{R_{w}x_{1}}{L_{w}}-\frac{x_{5}\left(1-\mu_{1}\right)}{L_{w}}-{I_{w}}^{*}+\eta_{1}e_{1}+\lambda_{1}\theta_{1}e_{1}{\left(\int_{0}^{t}e_{1}\cdot dt\right)}^{\theta_{1}-1}.$$

It can be observed from (21) that the control law comprised of two terms, i.e., the equivalent control $u_{e}$, which pushes the system toward the sliding surface, and the switching term $u_{s}$, which maintains the system on the surface under variation in the system parameters and external disturbances. For ease of control design, the control law can be defined as,

(22) $$u=u_{e}+u_{s}.$$

To derive $u_{e}$, substituting $\dot{S_{1}}=0$ in (21), we obtain:

(23) $$u_{e1}=1-\frac{L_{w}}{x_{5}}\left[\frac{V_{w}}{L_{w}}-\frac{R_{w}x_{1}}{L_{w}}-{I_{w}}^{*}+\eta_{1}e_{1}+\lambda_{1}\theta_{1}e_{1}{\left(\int_{0}^{t}e_{1}\cdot dt\right)}^{\theta_{1}-1}\right].$$

Furthermore, the switching term $u_{s1}$ can be defined as:

(24) $$u_{s1}=-\psi_{1}sign\left(S_{1}\right),$$

where $\psi_{1}$ is an exponential reaching law (ERL) and can be given as:

(25) $$\psi_{1}=\frac{k_{1}{\left|S_{1}\right|}^{\alpha_{1}}}{L(S_{1})}sign\left(S_{1}\right),$$

where,

(26) $$L\left(S_{1}\right)=\delta_{1}+\left(1-\delta_{1}\right)e^{-\rho_{1}{\left|S_{1}\right|}^{b_{1}}}.$$

The ERL increases the effectiveness of the control law by accelerating convergence and reducing chattering. The control law also functions effectively whether the system is far from or close to a sliding surface. The constraints on the parameters defined in (26) are: $k_{1}>0$, $\rho_{1}>0$, $0<\alpha_{1}<1$, and $0<\delta_{1}<1$. $\left|S_{1}\right|$ represents the mean value of $S_{1}$. In case the system states are far from the sliding surface, the first term in (26) accelerates the convergence of the states to the sliding surface. Once the system states are near the surface, the latter term ensures quicker convergence at the sliding surface. Thus, ERL enables the system to demonstrate a quick transient response under different scenarios of the reaching process by combining these two concepts. Similar sliding surfaces are defined for the errors $e_{2}$ and $e_{3}$ as follows:

(27) $$\left[\begin{array}{c} S_{2}\\ S_{3} \end{array}\right]=\left[\begin{array}{c} e_{2}+\eta_{2}\int_{0}^{t}e_{2}\cdot dt+\lambda_{2}{\left(\int_{0}^{t}e_{2}\cdot dt\right)}^{\theta_{2}}\\ e_{3}+\eta_{3}\int_{0}^{t}e_{3}\cdot dt+\lambda_{3}{\left(\int_{0}^{t}e_{3}\cdot dt\right)}^{\theta_{3}} \end{array}\right].$$

Taking the derivative of sliding surfaces $S_{2}$ and $S_{3}$ with respect to time yields:

(28) $$\left[\begin{array}{c} \dot{S_{2}}\\ \dot{S_{3}} \end{array}\right]=\left[\begin{array}{c} \dot{e_{2}}+\eta_{2}e_{2}\cdot dt+\lambda_{2}\theta_{2}e_{2}{\left(\int_{0}^{t}e_{2}\cdot dt\right)}^{\theta_{2}-1}\\ \dot{e_{3}}+\eta_{3}e_{3}\cdot dt+\lambda_{3}\theta_{3}e_{3}{\left(\int_{0}^{t}e_{3}\cdot dt\right)}^{\theta_{3}-1} \end{array}\right].$$

Utilizing (15)–(17) and (19), the sliding surface dynamics can be yielded as:

(29) $$\left[\begin{array}{c} \dot{S_{2}}\\ \dot{S_{3}} \end{array}\right]=\left[\begin{array}{c} \frac{I_{pv}}{C_{dc}}-\frac{\mu_{2}x_{3}}{C_{dc}}-\dot{V_{pv}^{*}}+\eta_{2}e_{2}\cdot dt+\lambda_{2}\theta_{2}e_{2}{\left(\int_{0}^{t}e_{2}\cdot dt\right)}^{\theta_{2}-1}\\ \frac{V_{b}}{L_{b}}-\frac{R_{b}x_{4}}{L_{b}}-\frac{x_{5}\mu_{34}}{L_{b}}-\dot{I_{b}^{*}}+\eta_{3}e_{3}\cdot dt+\lambda_{3}\theta_{3}e_{3}{\left(\int_{0}^{t}e_{3}\cdot dt\right)}^{\theta_{3}-1} \end{array}\right].$$

Based on the aforementioned control design procedure, ERL-GTSMC control laws have been derived for PVS and battery systems.

(30) $$u_{e2}=\frac{C_{dc}}{x_{3}}\left[\frac{I_{pv}}{C_{dc}}-\dot{{V_{pv}}^{*}}-\eta_{2}e_{2}-\lambda_{2}\theta_{2}e_{2}{\left(\int_{0}^{t}e_{2}\cdot dt\right)}^{\theta_{2}-1}\right],$$

(31) $$u_{e34}=\frac{L_{b}}{x_{5}}\left[\frac{V_{b}}{L_{b}}-\frac{R_{b}x_{3}}{L_{b}}-\dot{{I_{b}}^{*}}+\eta_{3}e_{3}+\lambda_{3}\theta_{3}e_{3}{\left(\int_{0}^{t}e_{3}.dt\right)}^{\theta_{3}-1}\right].$$

Similarly, the $u_{s}$ term can be derived as:

(32) $$u_{s2}=-\psi_{2}sign\left(S_{2}\right),$$

(33) $$u_{s34}=-\psi_{3}sign\left(S_{3}\right),$$

where $\psi_{2}$ and $\psi_{34}$ are the ERLs and can be stated as:

(34) $$\psi_{2}=\frac{k_{2}{\left|S_{2}\right|}^{\alpha_{2}}}{\delta_{2}+\left(1-\delta_{2}\right)e^{-\rho_{2}{\left|S_{2}\right|}^{b_{2}}}}sign\left(S_{2}\right).$$

(35) $$\psi_{3}=\frac{k_{3}{\left|S_{3}\right|}^{\alpha_{3}}}{\delta_{3}+\left(1-\delta_{3}\right)e^{-\rho_{3}{\left|S_{3}\right|}^{b_{3}}}}sign\left(S_{3}\right).$$

The constraints on the control parameters defined in $\psi_{2}$ and $\psi_{3}$ have been elaborated in (26). Furthermore, to provide the asymptotic stability of the proposed wind/PV/battery DC microgrid system, Lyapunov stability criteria have been utilized. According to the Lyapunov stability criteria, the candidate function for the sliding surface $\left(S_{1}\right)$ can be defined as:

(36) $$V\left(S_{1}\right)=\frac{1}{2}{S_{1}}^{2}.$$

To ensure the asymptotic stability of the system, $V(S_{1})$ should be positive definite and its time derivative should be negative definite, i.e., V(0)=0 and $\dot{V(S_{1})\leq 0}$.

(37) $$\begin{array}{cc} \dot{V}\left(S_{1}\right)= & S_{1}\dot{S_{1}}\\ = & S_{1}\left[\frac{V_{w}}{L_{w}}-\frac{R_{w}x_{1}}{L_{w}}-\frac{x_{5}\left(1-\mu_{1}\right)}{L_{w}}\right.\\ \; & \left.-{I_{w}}^{*}+\alpha_{1}e_{1}+\lambda_{1}\theta_{1}e_{1}{\left(\int_{0}^{t}e_{1}\cdot dt\right)}^{\theta_{1}-1}\right]\\ = & S_{1}\left[\frac{V_{w}}{L_{w}}-\frac{R_{w}x_{1}}{L_{w}}-\frac{x_{5}\left(1-\left(u_{e1}+u_{s1}\right)\right)}{L_{w}}\right.\\ \; & \left.-{I_{w}}^{*}+\alpha_{1}e_{1}+\lambda_{1}\theta_{1}e_{1}{\left(\int_{0}^{t}e_{1}\cdot dt\right)}^{\theta_{1}-1}\right]\\ = & -\psi_{1}\cdot \frac{x5}{L_{w}}\cdot S_{1}\cdot sign\left(S_{1}\right)\\ = & -\psi_{1}\cdot \frac{x5}{L_{w}}\cdot \left|S_{1}\right|\\ \leq & 0 \end{array}$$

Based on a similar design procedure, the asymptotic stability of $S_{2}$ and $S_{3}$ can also be achieved.

## 4. Energy Management System (High-Level)

An Energy Management System (EMS) has been devised to ensure power balance and provide the required reference values for the battery system. The EMS has two operational modes: surplus mode and shortage mode. The supervisory controller switches to surplus mode when the power produced by PVS and WES is greater than the load demand. On the other hand, a shortage mode is engaged when the produced power is lower than the demand. The objectives of the EMS are: (1) to ensure the smooth operation of the DC microgrid, (2) to extend the lifetime of the battery, and (3) to keep the battery within its designated State of Charge (SOC) range.

Shortage Mode: If $P_{balance}>0$, the EMS switches to this mode, and the battery supports PV and wind to cater to the load demands. The process is initiated by checking the battery SOC. If the SOC is greater than $SOC_{\min}=20\%$ for steady operation, the battery provides power. The battery will keep providing power until the SOC falls below its threshold. At this point, the battery system will be shut down, and load shedding will be applied to keep the DC microgrid operating steadily.

**Figure 7:**
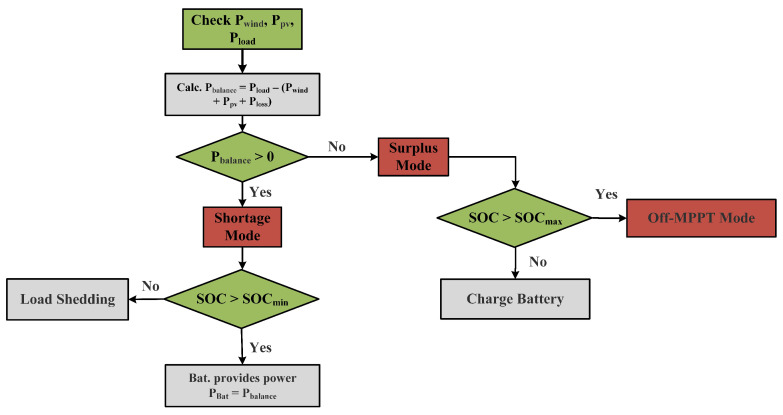
Flowchart of EMS algorithm.

Surplus Mode: If $P_{balance}<0$, the EMS switches to surplus mode. In this mode, the excess power is supplied for battery charging since the load demand is lower than the power produced by solar and wind energy sources. If the battery SOC is less than 80% $\left(SOC<SOC_{\max}\right)$, the EMS will charge the battery on a priority basis. If the battery SOC is greater than 80% $\left(SOC>SOC_{\max}\right)$, the battery charging will be stopped, and the renewable sources will switch to off-MPPT mode to stop wastage of power, as shown in Figure 7.

## 5. Results and Discussion

**Figure 8:**
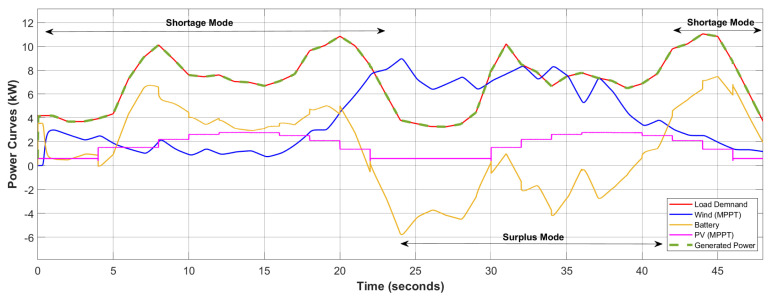
Power generation and demand.

**Figure 9:**
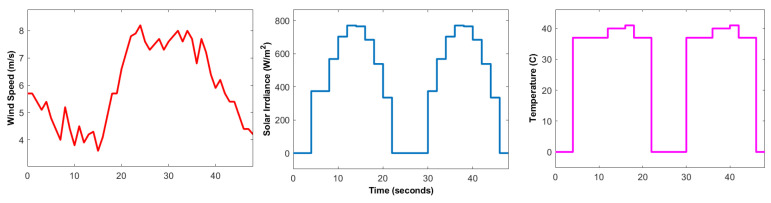
Varying wind speed, solar irradiance and temperature.

The robustness of the two-step control design based on DNN and ERL-GTSMC is assessed by running MATLAB simulations and C-HIL tests. The MATLAB/Simulink environment is used to implement the governing system model, which includes the WES, PVS, battery, power converters, and DC load. For accurate analysis, the ode45 solver with a step size of $10\times 10^{-5}$ is used. All the parameters and configuration of the designed DC microgrid and control laws are listed in Table A1 and Table A2. The performance of the suggested framework is examined under varying real-world load profiles and weather conditions (wind, temperature, solar irradiance). The load data are imported from OpenEI using the Pymgrid Python library and shown in Figure 8. It shows the 48-h profile of commercial and residential loads ranging from 2 kW to 12 kW. For simulation purposes, the load profile is scaled down to 48 s. A real-time wind profile is used for the MPPT of WES, as shown in Figure 9, to increase the accuracy of controller analysis. Similarly, varying solar irradiance and temperature are used for the MPPT of PVS. The wind speed information is gathered from an anemometer tower at the Jiangsu wind farm, which has a 50 MW capacity. The reference DC voltage value chosen for all simulations is 700 V.

**Figure 10:**
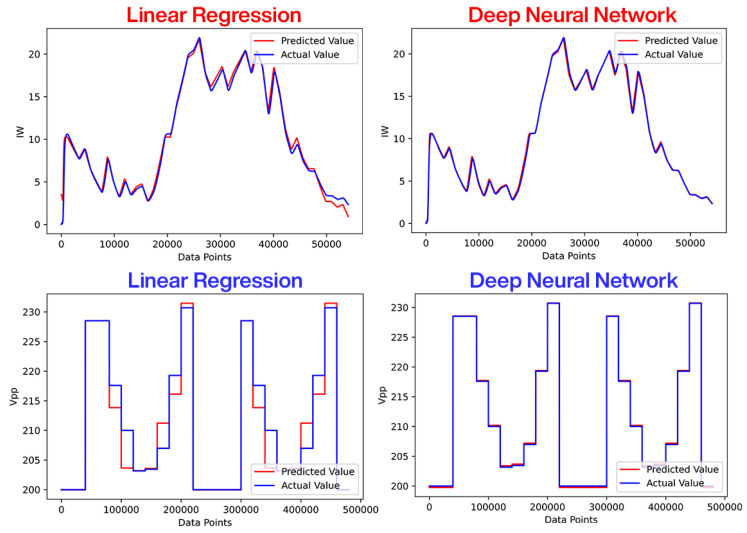
DNN based MPPT reference generation for WES and PVS.

The MPPT references for the WES and PVS are generated using DNN and compared with the linear regression technique, as shown in Figure 10. It can be noticed that DNN predicts the actual value more accurately compared to linear regression in both cases.

**Figure 11:**
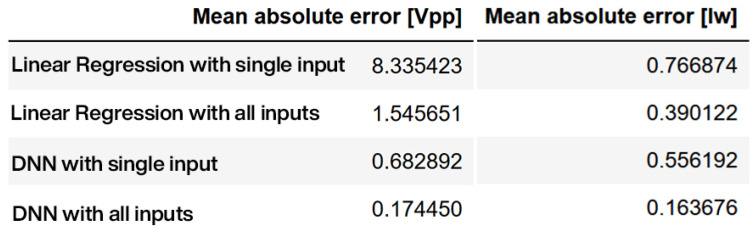
Linear regression and DNN performance analysis.

To measure efficiency, the mean absolute error (MAE) is used as a comparison parameter. MAE calculates the average amount of error. As a result, the machine learning model with the lowest MAE should be considered a good model. From the comparison given in Figure 11, it can be observed that DNN single input models have lower MAE values of 0.68 and 0.55 compared to 8.33 and 0.76 in the case of linear regression. Similarly, with multiple inputs, DNN models perform better with an MAE of 0.17 and 0.16 compared to 1.54 and 0.39 in the case of linear regression.

**Figure 12:**
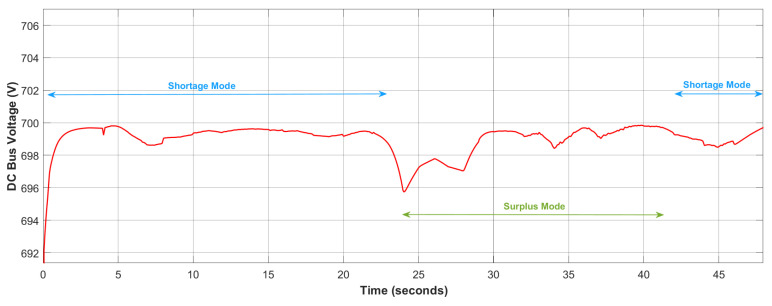
Regulation of DC bus voltage.

The effectiveness of the proposed two-step controller in rigorously stabilizing the DC bus voltage against perturbations in load demands and environmental parameters (wind speed, solar irradiance, and temperature) has been shown in Figure 12. From Figure 12, it is clear that over the entire simulated time window, $V_{dc}$ has been regulated to the 700 V reference value. The proposed ERL-GTSMC demonstrates a quick response, reaching the steady state with a settling time of 0.05816 s. The controller also achieves the objective of minimizing chattering and reducing undershoot and overshoot. As shown in Figure 12, the stability of the DC bus voltage was also evaluated in both surplus and shortage modes, and the accurate tracking of the ERL-GTSMC controller was observed with a steady-state error of only 1.1 V.

**Figure 13:**
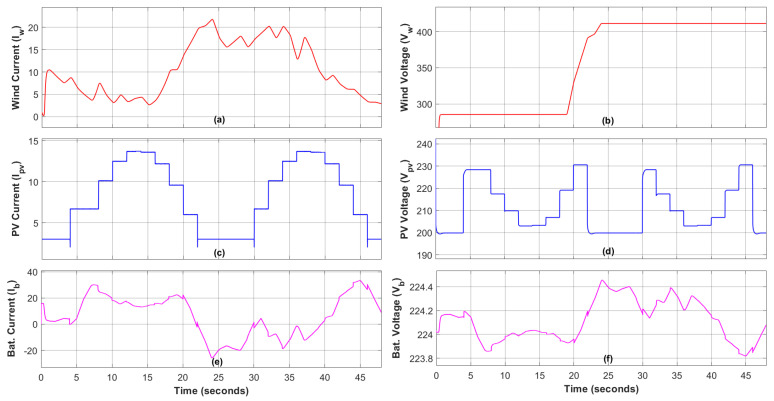
Current and voltage of power generation units: (**a**) Wind Current, (**b**) Wind Voltage, (**c**) PV Current, (**d**) PV Voltage, (**e**) Battery Current, (**f**) Battery Voltage.

The WES, PVS, and battery system voltage and current levels have been presented in Figure 13. The DNN-based reference generation and the tracking of reference current and voltage by ERL-GTSMC have been shown in Figure 13a,b. From 15 s to 37 s, it can be deduced that the generated current rises with respect to wind speed; however, when the wind speed becomes slow at 37 s, the generated current falls with respect to it. Figure 13c,d depicts the PVS current and voltage curves to achieve MPPT using DNN and ERL-GTSMC. It can be noticed that as the solar irradiance increases from 0 W/m${}^{2}$ to 386 W/m${}^{2}$ at 4 s, the generated power also increases. Similarly, the power peaks at 12 s when the solar irradiance is at the highest level of 780 W/m${}^{2}$. The current and voltage of the battery are shown in Figure 13e,f. From 0 s to 20 s, the battery discharges to compensate for power shortage, and when the power demand is lower than the generated power, the surplus power is supplied to the battery for charging.

Figure 8 depicts the power curves of WES, PVS, the battery, total load generation, and load demand. Initially, the load demand is low, but the renewable sources are also generating very little power. Therefore, the EMS operates in shortage mode, and the battery supports the microgrid by discharging. At 5 s, the load demand starts to gradually rise, but the system is still functioning in shortage mode. To accommodate the increase in load demand, the depletion in the battery system can be seen at 5 s. At 22 s, due to a decrease in load demand and an increase in renewable generation, the EMS switches to surplus mode, and the excess power is used to charge the battery. At 41 s, the load demand once again exceeds the power generated by renewables, so the battery switches to discharging mode and supports renewable generation.

**Figure 14:**
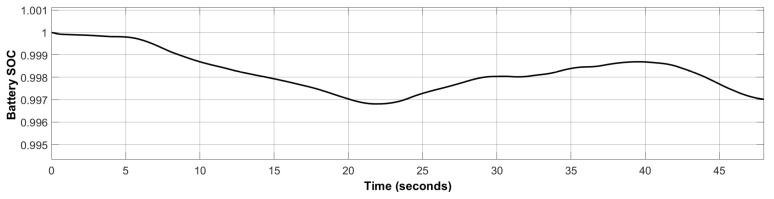
Battery SOC.

The charging/discharging characteristic of the battery is demonstrated in Figure 14. When the generated power is insufficient to supply the load demand and SOC ≥ $SOC_{min}$, the battery, along with PVS and WES, provides the energy deficit to meet the load demand. From 20 to 40 s, the battery is charged in accordance with the EMS instructions as shown in Figure 7.

### 5.1. Comparative Analysis with Traditional Control Methods

**Figure 15:**
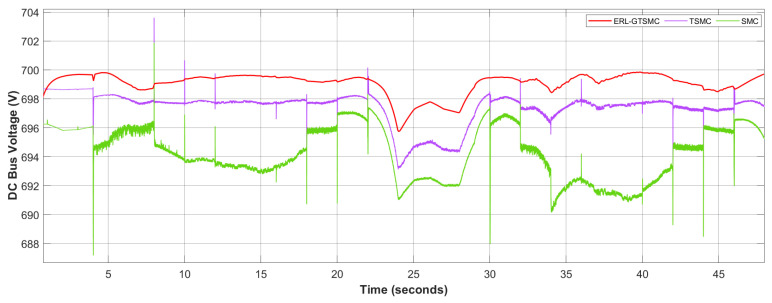
DC bus voltage Tracking.

The designed ERL-GTSMC controller is compared to traditional TSMC and SMC in terms of settling time, chattering phenomenon, steady-state error, and overshoot. To ensure a fair analysis, the same tuning configuration and simulation environment are used. The tracking of DC bus voltage, battery current, WES current, and PVS current is investigated to assess the robustness, reaching speed, and transient response of the controllers. Figure 15 compares the DC bus regulation performance of ERL-GTSMC to that of TSMC and SMC. It can be noticed that ERL-GTSMC has a good settling time of 0.05816 s, but SMC is the fastest with a settling time of 0.0317 s. However, the proposed controller shows a good response to the transients and shows zero overshoot and undershoot, while in the case of SMC and TSMC, an overshot of 1.82% and 0.714% is observed, respectively. Furthermore, ERL-GTSMC has superior tracking accuracy and exhibits a steady-state error (SSE) of 0.0571%, while in the case of SMC and TSMC, it is 0.971% and 0.314%, respectively. Finally, ERL-GTSMC significantly reduces the chattering phenomenon and shows very negligible chattering. However, high chattering is observed in the case of SMC and TSMC. Table 1 depicts the numerical comparison of the proposed ERL-GTSMC to that of TSMC and SMC.

### 5.2. Experimental Testing Using Controller Hardware-in-the-Loop (C-HIL) Setup

**Figure 16:**
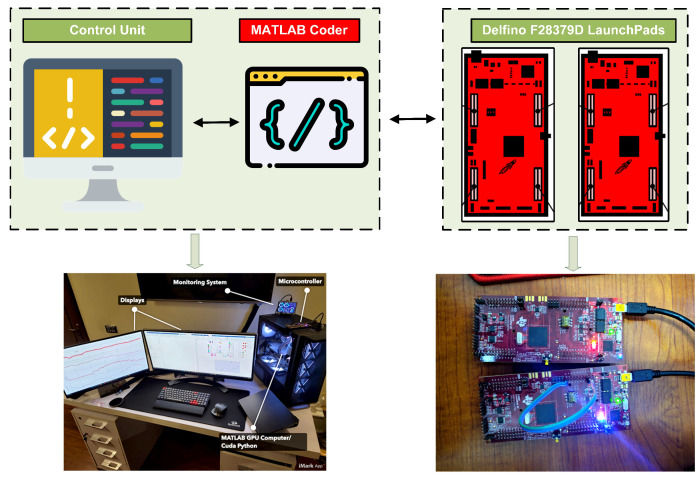
Configuration of the C-HIL.

The setup for real-time experimental testing of the proposed ERL-GTSMC using the TI LAUNCHXL-F28379D is shown in Figure 16. The TMS320F28379D dual-core processors used in this workbench operate at a frequency of 200 MHz. The development kit is interfaced with the control unit via the C2000 embedded coder package in MATLAB/Simulink. First, the mathematical model of the proposed DC microgrid framework is designed in MATLAB, and then the code is transferred to the F28379D development kit α. In addition, the ERL-GTSMC laws have been executed on development kit β to provide the required PWM signals to the transistor switches of the power converters at a switching frequency of 25 kHz. The development kit β’s PWM output ports are connected to development kit α’s GPIO ports using the available 12-bit ADC and DAC. A real-time closed-loop system has been established between launchpad α and β. To evaluate the results of C-HIL compared to simulated results, tests are conducted under varying wind speeds and load demands.

**Figure 17:**
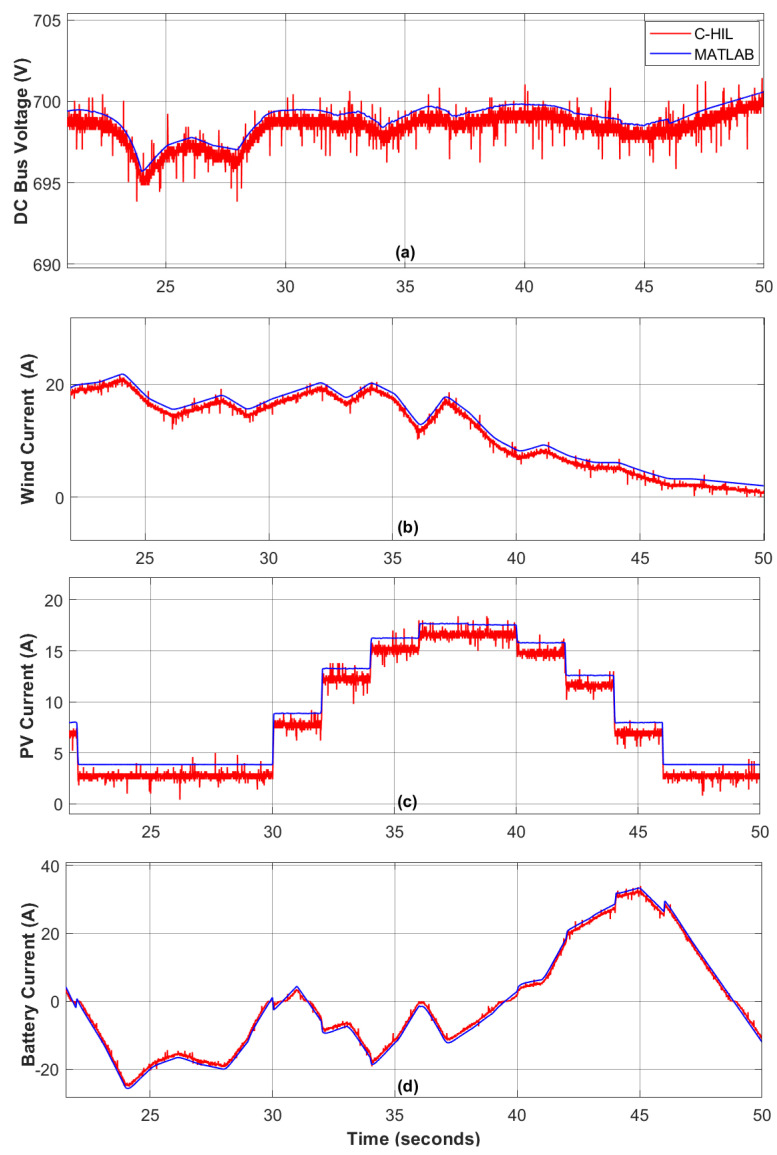
C-HIL Results. (**a**) DC Bus Voltage, (**b**) Wind Current, (**c**) PV Current, (**d**) Battery Current.

Figure 17 illustrates the C-HIL platform used to examine ERL-GTSMC controllers under both surplus and shortage modes. During C-HIL, the reference voltage for the DC bus voltage is set to 700 V, and the tuning parameters obtained during computer simulations are used for hardware testing. The DC bus dynamics are shown in Figure 17, and it can be observed that the voltage is strictly maintained during both surplus and shortage modes, with a tracking accuracy of 99.84%. Figure 17b shows the wind current profile under dynamic wind speed. The performance of PVS is depicted in Figure 17c. The charging and discharging state of the battery during surplus and shortage modes are shown in Figure 17d. It can be verified from the aforementioned controller analysis performed in a C-HIL environment that the ERL-GTSMC controllers have adequate tracking and quick response to transients. Furthermore, in every situation, the performance of C-HIL is as robust as the performance of the computer simulation.

## 6. Conclusions

DNN-based MPPT reference generation and global terminal sliding model control with the exponential reaching law were presented for a DC microgrid. The DC microgrid bus and its components are mathematically modeled, and control laws are derived. The DC microgrid is extensively tested using real-world weather conditions and various operational modes (surplus mode, shortage mode). The proposed ERL-GTSMC demonstrated a quick response, with a settling time of 0.05816 s, and precise steady-state accuracy, with an SSE of just 0.0571%. Additionally, the proposed control technique exhibits a significant decrease in the chattering phenomenon that typically occurs in slide mode controllers. The proposed ERL-GTSMC was found to be superior in all circumstances compared to TSMC and SMC. The real-time performance of the suggested ERL-GTSMC was validated by the C-HIL process using a low-cost evaluation kit boosting a 200 MHz dual-core processor. Future work will include designing the ERL-GTSMC for a DC microgrid connected to the grid.

## Figures and Tables

**Table 1:** Comparison of proposed ERL-GTSMC with TSMC and SMC.

	Settling Time (s)	Chattering	Percent Overshoot (%)	Steady State Error (SSE) (%)
**SMC**	0.0317	High	1.82	0.971
**TSMC**	0.0445	Mild	0.714	0.314
**ERL-GTSMC**	0.05816	Low	0.00	0.0571

## Data Availability

Not applicable.

## Appendix A

### Appendix A.1

**Table A1:** Configuration of energy sources.

Wind Turbine/PMSG	
Density (air)	1.2 kg/m${}^{3}$
Wind speed	5.5 m/s
$\lambda_{op}$	7
$C_{pmax}$	0.43
Nominal RPM	1000
Line-line Voltage	400 V
Rated Power	20 kW
Battery	
Rated Voltage	230 V
Rated Battery Capacity	18 Ah
Batteries connected in series	3
Photovoltaic System	
Rated Voltage	229 V
Rated Current	14 A
Rated power of single module	2 kW
Rated Maximum Power	3.2 kW

### Appendix A.2

**Table A2:** Parameters of the overall system and its control.

DC Microgrid Configuration	
DC-link Voltage $V_{dc}$	700 V
Frequency (MOSFETs Switching)	25 kHz
Resistances $R_{w},R_{pv},R_{b}$	7 mΩ, 20 mΩ, 20 mΩ
Inductance $L_{w},L_{pv},L_{b}$	3.3 mH, 3.8 mH, 3.1 mH
Capacitance $C_{dc}$	68 μF
Control Parameters	
ERL-GTSMC	$\eta_{1}=1$, $\eta_{2}=1$, $\eta_{3}=2$, $\lambda_{1}=2$, $\lambda_{2}=2$
	$\lambda_{3}=1.2$, $k_{1}=160$, $k_{2}=445$, $k_{3}=200$
	$\theta_{1}=1.6$, $\theta_{2}=1.5$, $\theta_{3}=1.3$
TSMC	$\eta_{1}=1$, $\eta_{2}=1$, $\eta_{3}=2$, $\lambda_{1}=2$, $\lambda_{2}=2$
	$\lambda_{3}=1.1$, $\theta_{1}=1.6$, $\theta_{2}=1.5$, $\theta_{3}=1.3$
SMC	$K_{s1}=75$, $K_{s2}=100$, $K_{s3}=500$
